# Imaging Characteristics and Prognostic Value of Isolated Pulmonary Metastasis from Colorectal Cancer Demonstrated with^18^F-FDG PET/CT

**DOI:** 10.1155/2022/2230079

**Published:** 2022-04-14

**Authors:** Yu Yu, Jing Zhu, Yeye Zhou, Shibiao Sang, Yuchun Zhu, Xiaoyi Zhang

**Affiliations:** ^1^Department of Nuclear Medicine, The First Affiliated Hospital of Soochow University, Suzhou, China; ^2^Department of Nuclear Medicine, Changshu No. 2 People's Hospital, Changshu, China; ^3^Department of Nuclear Medicine, First People's Hospital of Kunshan, Kunshan, China

## Abstract

**Objective:**

Solitary pulmonary lesions (SPNs) in patients with a history of colorectal cancer (CRC) may be attributed to metastatic lung tumors, primary lung cancer, or benign nodules. We aimed to analyze the imaging characteristics of SPNs in CRC patients to differentiate these pulmonary nodules and evaluate the prognostic value of isolated pulmonary metastasis from CRC using ^18^F-FDG PET/CT.

**Methods:**

From January 2013 to January 2021, 62 CRC patients with SPNs demonstrated with ^18^F-FDG PET/CT were retrospectively enrolled in the present study. We compared the radiological and clinical characteristics of these patients. In addition, survival time and prognostic factors were statistically analyzed using the Kaplan-Meier method and multivariable Cox proportional hazards models.

**Results:**

There were 33 cases of isolated lung metastasis, 20 cases of second primary lung cancer (SPLC), and nine cases of benign nodules. The proportion of nodules with a maximal diameter greater than the median value was lower in the isolated lung metastasis group compared with the SPLC group (*p* < 0.05), showing polygonal shape, ill-defined margin, pleural indentation, air bronchogram, speculation, and ground-glass opacity. Patients with isolated lung metastasis had a significantly higher maximal diameter of lung lesion, SUV_max_ of lung lesion, and ^18^F-FDG uptake compared with the benign nodule group (*p* < 0.05). Multivariate analysis revealed that the following two factors were significant independent predictors of PLC: air bronchogram (hazard ratio [HR] =22.327; 95% confidence interval [CI]: 1.910-261.061; *p* = 0.013) and spiculation (HR =6.148; 95% CI 1.469-25.725; *p* = 0.013). Initial TNM stage IV (HR =19.831, 95% CI 1.061-370.782; *p* = 0.046) was extremely associated with a decreased lifespan of CRC patients with isolated lung metastasis.

**Conclusions:**

The result showed that CT features, including air bronchogram and spiculated margins, could be used to differentiate SPLC from single isolated lung metastasis in CRC patients. In patients with isolated lung metastasis, primary CRC TNM stage IV was associated with a poorer prognosis, and patients with such conditions might need more care.

## 1. Introduction

Colorectal cancer (CRC) ranks third in incidence and is the second leading cause of cancer-related mortality worldwide. Over 1.8 million new cases of CRC and 881,000 deaths are estimated to occur in 2018 [1]. Metastasis significantly contributes to treatment failure and cancer-associated death in CRC [2, 3]. Pulmonary metastasis is one of the most frequent extra-abdominal sites of CRC [4]. However, about 10% of pulmonary metastasis is presented as solitary pulmonary lesions (SPNs), which mimic primary lung cancer (PLC) [5]. Although surgery is always recommended for solitary pulmonary metastasis and PLC, the type of resection procedure may vary from limited resections in cases of solitary metastasis to extended resections with lymphadenectomy if the lesion is PLC [6, 7].

^18^F-fluorodeoxyglucose (^18^F-FDG) positron emission tomography (PET) in combination with CT has become a well-established imaging modality to characterize SPNs and improve CRC staging [8, 9]. However, the differences in imaging characteristics between PLC and SPN and the prognostic value of isolated pulmonary metastasis from CRC demonstrated with ^18^F-FDG PET/CT remain largely unclear.

In the present study, we aimed to analyze the imaging characteristics of SPNs in CRC patients to differentiate these pulmonary nodules and evaluate the prognostic value of isolated pulmonary metastasis from CRC demonstrated with ^18^F-FDG PET/CT.

## 2. Methods

### 2.1. Patient Selection

This study was approved by the institutional review board of the First Affiliated Hospital of Soochow University. Trial registration number: ChiCTR2100045115. Since the trial was a retrospective study, written informed consent for this study was waived by the ethics committee, and no personal information was disclosed. This study complied with the Declaration of Helsinki.

From January 2013 to January 2021, CRC patients with SPNs demonstrated with ^18^F-FDG PET/CT were retrospectively enrolled in the present study. The following patients were excluded: (1) patients had additional malignancies; (2) digital image data unavailable for retrospective analysis; (3) patients with appendiceal tumors; and (4) PET was performed in 6 weeks after the end of chemotherapy, and 3 months after any radiotherapy. If the size of the lesion was less than 5 mm, periodical CT surveillance was recommended until lesion growth was observed.

### 2.2. Clinicopathological Features and Response Evaluation

The following clinicopathological factors were evaluated: gender, age, initial tumor location, histological type, initial TNM stage, treatment of CRC, serum carbohydrate antigen19-9 (CA19-9) and carcinoembryonic antigen (CEA) levels at pulmonary nodule detection, interval to pulmonary nodules (ITP), and overall survival (OS). In addition, the radiological parameters on high-resolution CT (HRCT) included shape (polygonal, round), margin (well-defined, ill-defined), pleural indentation, calcification, cavity, air bronchogram, and ground-glass opacity (GGO) (present or absent).

### 2.3. Image Acquisition

PET/CT images were acquired at 60 ± 5 min after injection of ^18^F-FDG (dose 0.12 mCi/kg) according to the European Association of Nuclear Medicine (EANM) guidelines [10]. All patients were subjected to food deprivation for 6 h before the 18F-FDG PET/CT (General Electric Medical Systems, Milwaukee, WI, USA). The baseline blood glucose level was lower than 11 mmol/L. Attenuation-corrected images were acquired via low-dose CT (140 kV; 120 mA). Emission PET data were obtained after CT scanning, with 2-3 min/bed position. PET and CT images were reconstructed using a standard iterative algorithm (ordered-subset expectation maximization). Thin-section CT (TSCT) parameters were set as follows: 140 kV, 120 mA, 0.8 s rotation time thickness, 700 mm field of view, and 3.75 mm slice thickness. An ADW4.1 workstation (GE Healthcare) was used to display whole-body PET/CT images.

^18^F-FDG PET/CT images were independently evaluated by two experienced nuclear medicine physicians who were blinded to the clinical information of all the subjects, and the results were recorded by consensus. ^18^F-FDG uptake was evaluated using SUV_max_, and the highest FDG uptake was considered the SUV_max_ of the lung lesion. SUV_max_ ≥ median was classified as PET-positive.

### 2.4. Statistical Analysis

All statistical analyses were performed using the GraphPad Prism 5.0 software (GraphPad Software Inc., San Diego, CA, USA) and IBM SPSS 19.0 software (SPSS Inc., Chicago, IL, USA). ITP was defined as the period between the diagnosis of CRC and the identification of SPNs. OS was defined as the duration between the diagnosis and the final follow-up or all-cause mortality. The clinicopathological characteristics of the groups were compared using Pearson's chi-square test. The survival rates were estimated with the Kaplan-Meier method, and differences among groups were evaluated by a log-rank test. Univariate analysis for OS-associated factors was performed. Risk factors with statistical significance upon univariate analysis were introduced into a Cox proportional hazards model for multivariate analysis. A *p* value of <0.05 was considered statistically significant.

## 3. Results

### 3.1. Clinical Characteristics

Table 1 summarizes the detailed clinical characteristics of CRC patients diagnosed with SPNs. A total of 62 CRC patients (45 males and 17 females; mean age 66 [37-83] years) were eligible to analyze SPNs in this study. Of these patients, there were 33 cases of isolated lung metastasis, 20 cases of second PLC (SPLC), and nine cases of benign nodules. The median follow-up period for all patients was 20 (2-84) months.

**Figure 1:**
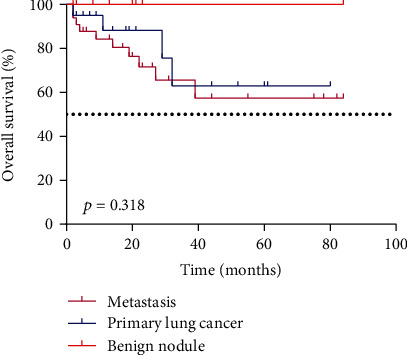
Kaplan-Meier survival analysis of the OS of CRC patients with isolated pulmonary nodules. The dotted line represents a 50% survival rate.

Except that there were more male patients in the isolated lung metastasis group compared with the SPLC group (*p* = 0.035), other factors, including age, primary CRC tumor location, initial TNM stage, histological type, treatment of CRC, serum CEA level, serum CA19-9 level, ITP, and OS, were not significantly different among the three groups (*p* > 0.05; Figure 1).

### 3.2. PET/CT Imaging Features of SPNs

Table 2 demonstrates the imaging features of the SPNs in all patients. All patients had solid SPNs (mean diameter: 23.85 ± 13.84 mm, range: 5-60 mm) that were suspected to be malignant. The lesions were centrally located in 14 (22.58%) patients and peripherally located in 48 (77.42%) patients. On ^18^F-FDG PET/CT, the median SUV_max_ of all nodules was 3.87. The mean SUV_max_ in all nodules was 5.36 ± 4.21.

The maximal diameters of the nodules, lesion location, SUV_max_ of lung lesion, ^18^F-FDG uptake, calcification, cavity, and presence of enlarged mediastinal lymph nodes, or other sites of metastasis were not significantly different between the patients with isolated lung metastasis and those with SPLC (*p* > 0.05). However, the proportion of nodules with a maximal diameter greater than the median value was lower in the isolated lung metastasis group compared with the SPLC group (*p* < 0.05), showing polygonal shape, ill-defined margin, pleural indentation, air bronchogram, speculation, and ground-glass opacity.

We also compared the radiological characteristics of CRC patients with isolated lung metastasis and benign nodules. Patients with isolated lung metastasis had a significantly higher maximal diameter of lung lesion, SUV_max_ of lung lesion, and ^18^F-FDG uptake (*p* < 0.05). GGO component was more frequently detected in benign nodules compared with SPNs.

### 3.3. Multivariate Analysis for the Factors That Distinguish PLC from Isolated Lung Metastasis

**Figure 2:**
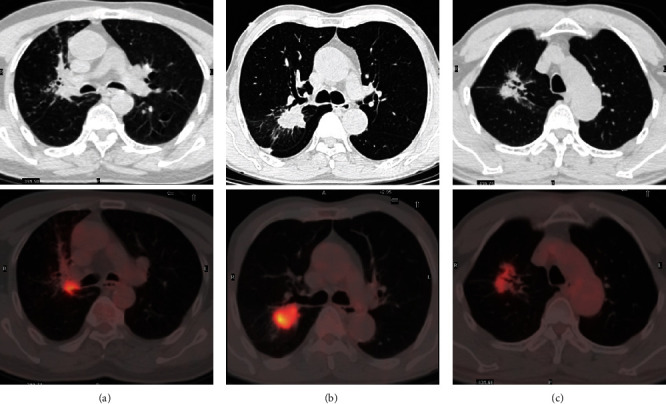
^18^F-FDG PET/CT findings of pulmonary lesions of PLC (a), atypical pulmonary metastasis of CRC (b), and atypical benign nodule (c). (a) A 68-year-old man with a history of rectal carcinoma. ^18^F-FDG PET/CT revealed a pulmonary lesion with air bronchogram (SUV_max_, 8.4; D_max_, 24 mm). The patient underwent pulmonary surgery. Pathological examination confirmed PLC. (b) A 64-year-old man with a history of rectal carcinoma. ^18^F-FDG PET/CT revealed a pulmonary lesion that was suspected to be malignant (SUV_max_, 9.93; D_max_, 32 mm). Pulmonary surgery confirmed pulmonary metastasis. (c) A 62-year-old man with a history of rectal carcinoma.^18^F-FDG PET/CT revealed a pulmonary lesion that was suspected to be malignant (SUV_max_, 3.84; D_max_, 14 mm), pleural indentation, and spiculation. Pulmonary surgery confirmed inflammatory disease.

Table 3 shows the results of multivariate analysis for the clinical and radiological factors that could discriminate between isolated lung metastasis and SPLC. Multivariate analysis revealed that the following two factors were significant independent predictors of PLC: air bronchogram (hazard ratio [HR] =22.327; 95% confidence interval [CI]: 1.910-261.061; *p* = 0.013) and spiculation (HR =6.148; 95% CI 1.469-25.725; *p* = 0.013) (Figure 2).

### 3.4. Univariate and Multivariate Analyses of OS in the Isolated Lung Metastasis Group

Table 4 demonstrates that the maximal diameter and the ^18^F-FDG uptake of lung lesions did not affect the prognosis of CRC patients with isolated lung metastasis. Adjuvant chemotherapy of CRC was associated with a better prognosis, while hilar and/or mediastinal lymph node metastasis, air bronchogram component, initial TNM stage IV, CA19-9 level, and ITP<12 months were all significantly related to a worse prognosis in CRC patients with isolated lung metastasis (Figure S1).

Furthermore, the multivariate Cox proportional hazards model indicated that initial TNM stage IV (HR =19.831, 95% CI 1.061-370.782; *p* = 0.046) significantly contributed to the reduced lifespan of CRC patients with isolated lung metastasis (Figure S2).

## 4. Discussion

Isolated lung metastasis is more frequently reported in CRC patients than in those with other extrathoracic malignancies [5, 11, 12]. It is sometimes difficult to determine whether an SPN is PLC or an isolated lung metastasis because SPLC occasionally mimics isolated lung metastasis of CRC patients [13, 14]. However, different surgical strategies are adopted to treat these two lung nodules. In general, the gold standard for SPLC is the complete surgical resection with lobectomy and mediastinal lymph node dissection. In contrast, the treatment of lung metastasis is minimally invasive surgical resection to preserve healthy lung parenchyma in case a repeat operation is needed [15, 16]. Therefore, it is essential to distinguish between PLC and isolated lung metastasis before surgical planning.

In the present study, we investigated the feasibility of using PET/CT to differentiate PLC and isolated lung metastatic lesions in CRC patients. Air bronchogram and spiculated margins were beneficial discriminatory factors in multivariate analyses, which was in line with some previous studies [17–20]. As we know, a smooth or well-defined margin is more common in metastatic nodules than an irregular margin, while PLC usually presents with a non-smooth margin, especially a speculated margin [21, 22]. However, the margin of a nodule is more irregular when the size of isolated lung metastasis is increased [23]. Ohtaki et al. have reported that the presence or absence of GGO and pleural indentation can be helpful factors to distinguish between PLC and lung metastasis from CRC [24]. In their study, patients with more than one SPN are also included. Similarly, the proportion of GGO and pleural indentation in the isolated lung metastasis group was significantly higher compared with the PLC group in our univariate analysis. However, in multivariate analysis, they were not significant factors. A possible selection bias of patients suggested that these results should be interpreted with caution.

In terms of the prognosis of CRC patients with pulmonary tumors, survival differences were observed in the present work. Our data showed that OS was not significantly different between the isolated lung metastasis group and the PLC group. Chang et al. have reported that the median OS of CRC patients with PLC is longer than that of CRC patients with metastatic lung lesions, while the between-group difference is not significant. They believe that CRC patients with SPLC are diagnosed in the early clinical stage because of regular surveillance imaging. Early detection and immediate surgical resection may prolong survival and improve prognosis [25]. However, Ohtaki et al. have found that the outcome of CRC patients with lung metastasis is significantly better than the patients with radiologically solid lung cancer [24]. In their opinion, the introduction of less invasive surgical techniques, perioperative management, and various types of chemotherapy has improved the survival of CRC patients with lung metastasis. This controversy may be attributed to the selection bias of patients. In the present study, only CRC patients with SPNs were analyzed.

Consistent with previous reports [26, 27], we also found that the TNM stage of primary CRC significantly impacted survival. Nonetheless, there was no definite agreement on the value of its prognostic role. Furthermore, some authors fail to confirm a correlation between the primary TNM stage and survival [28, 29]. This discrepancy may be explained by different criteria for patient selection and treatment of primary CRC.

^18^F-FDG PET/CT has been shown to have high sensitivity and specificity for detecting mediastinal and hilar lymph node metastases [30]. According to our data, patients with concomitant mediastinal and/or hilar lymph node metastases are more likely to be diagnosed with PLC than metastatic lung tumors.

In the present study, we also analyzed the prognostic values of factors about PET/CT imaging, such as metastasis detected on PET/CT, maximal diameter of lung lesion, and SUV_max_. Only hilar and/or mediastinal lymph node metastasis were related to a worse prognosis in CRC patients with isolated lung metastasis in univariate analyses. Other factors were not found to be significant in this study. Moreover, some previous studies have shown that the presence of hilar or mediastinal lymph node metastases can predict a poor prognosis for CRC patients with lung metastasis [27, 28], and our results supported these results.

This study has several limitations. First, this was a retrospective study with a relatively small sample size. To the best of our knowledge, although this study was the largest single-center cohort of CRC patients with SPNs, the number of the cases was still small. Because the incidence of SPLC and single isolated lung metastasis in CRC patients is relatively low, such small sample numbers may lead to difficulty in statistical comparison. A prospective study and a larger cohort are needed to confirm our results. Second, the median follow-up time was 20 months, which was inadequate. More than 5 years of follow-up is required to confirm the prognostic value of isolated pulmonary metastasis from CRC demonstrated with ^18^F-FDG PET/CT. Third, CT features were visually analyzed in this study, which might raise the possibility of inter-observer and intra-observer variability despite consensus reading. More quantitative analysis tools, such as radiomics, would be more helpful for a more accurate interpretation. Fourth, the range of tumor size in this study was relatively large. On the one hand, small tumors are usually featured with well-defined margins, which are easily differentiated. On the other hand, small tumors may cause the inaccuracy of SUV_max_ value because of the degree of respiratory motion.

In conclusion, CT features, including air bronchogram and spiculated margins, could be used to differentiate between SPLC and single isolated lung metastasis in CRC patients. In patients with isolated lung metastasis, primary CRC TNM stage IV was associated with a poorer prognosis, and patients with such conditions might need more care.

## Figures and Tables

**Table 1:** Clinical characteristics of the 62 patients with isolated lung nodules.

Characteristic	All (*n* =62) (%)	Metastasis (*n* =33) (%)	Primary lung cancer (*n* =20) (%)	Benign nodule (*n* =9) (%)	*p*-value^a^	*p*-value^b^
Gender					**0.035**	0.866
Male	45 (72.58)	21 (63.6)	18 (90.0)	6 (66.7)		
Female	17 (27.42)	12 (36.4)	2 (10.0)	3 (33.3)		
Age (year)					0.629	0.554
Mean ±	65.02 ±	66.27 ±	65 ± 7.81	60.44 ±		
SD	9.44	10.01		10.21		
Primary tumor (%)					0.547	0.565
Rectum	34 (54.84)	17 (51.5)	12 (60.0)	5 (55.6)		
Colon	28 (45.16)	16 (48.5)	8 (40.0)	4 (44.4)		
Initial TNM stage					0.765	0.312
I-III	53 (85.48)	27 (81.8)	17 (85.0)	9 (100)		
IV	9 (14.52)	6 (18.2)	3 (15.0)	0 (0.0)		
Histological type					0.388	0.05
WD	1(1.61)	1 (3.03)	0 (0.00)	0 (0.0)		
MD	41 (66.13)	22 (66.67)	15 (75.00)	4 (15.4)		
PD	11 (17.74)	8 (24.24)	2 (10.00)	1 (11.1)		
Unknown	9 (14.52)	2 (6.06)	3 (15.00)	4 (66.7)		
Treatment of CRC						
Adjuvant chemotherapy					0.305	0.454
+	29 (46.77)	18 (54.5)	8 (40.0)	3 (33.3)		
-	33 (53.23)	15 (45.5)	12 (60.0)	6 (66.7)		
Adjuvant radiotherapy					0.512	0.475
+	4 (6.45)	3 (9.1)	1 (5.0)	0 (0.0)		
-	58 (93.55)	30 (90.9)	19 (95.0)	9 (100)		
CEA (ng/ml) level					0.472	0.253
≥5	18 (29.03)	12 (36.4)	5 (25.0)	1 (11.1)		
<5	40 (64.52)	20 (60.6)	13 (65.0)	7 (77.8)		
Unknown	4 (6.45)	1 (3.0)	2 (10.0)	1 (11.1)		
CA19-9 (U/ml) level					0.349	0.846
≥37	7 (11.29)	5 (15.2)	1 (5.0)	1 (11.1)		
<37	49 (79.03)	26 (78.8)	16 (80.0)	7 (77.8)		
Unknown	6 (9.68)	2 (6.1)	3 (15.0)	1 (11.1)		
ITP (months)						
≥3/<3 months	42 (67.74)/20 (32.26)	25 (75.8)/8 (24.2)	11 (55.0)/9 (45.0)	6 (66.7)/3 (33.3)	0.117	0.582
≥6/<6 months	41 (66.13)/21 (33.87)	25 (75.8)/8 (24.2)	11 (55.0)/9 (45.0)	5 (55.6)/4 (44.4)	0.117	0.44
≥12/<12 months	33 (53.23)/29 (46.77)	18 (54.5)/15 (45.5)	10 (50.0)/10 (50.0)	5 (55.6)/4 (44.4)	0.748	0.629
≥24/<24 months	24 (38.71)/38 (61.29)	10 (30.3)/23 (69.7)	9 (45.0)/11 (55.0)	5 (55.6)/4 (44.4)	0.279	0.313
Overall survival (months)					0.492	0.147
Median	Not reached	Not reached	Not reached	Not reached		

WD: well-differentiated; MD: moderately differentiated; PD: poorly differentiated; CEA: carcinoembryonic antigen; CA19-9: carbohydrate antigen 19-9; ITP: interval to pulmonary nodules. ^a^Test for difference between the isolated lung metastasis group and the second primary lung cancer group. ^b^Test for difference between the isolated lung metastasis group and the benign nodule group. Statistically significant *p*-values are highlighted in bold.

**Table 2:** Image characters of the isolated lung nodules in CRC patients.

Characteristic	Metastasis (*n* =33) (%)	Primary lung cancer (*n* =20) (%)	Benign nodule (*n* =9) (%)	*p*-value^a^	*p*-value^b^
Maximal diameter of lung lesion (mm)					
Median (range)	18 (5-55)	30 (13-60)	14 (9-27)	0.668	**0.045**
Comparison with the median value				**0.021**	0.472
≥21.65	14 (42.4)	15 (75.0)	2 (22.2)		
<21.65	19 (57.6)	5 (25.0)	7 (77.8)		
Lesion location				0.294	0.614
Central	6 (18.2)	7 (35.0)	1 (11.1)		
Peripheral	27 (81.8)	13 (65.0)	8 (88.9)		
SUV_max_ of lung lesion				0.165	**0.001**
Median (range)	4.27 (0.76-16.36)	6.7 (0.80-16.53)	2.4 (0.8-3.84)		
^18^F-FDG uptake				0.862	**0.002**
Positive	19 (57.6)	12 (60.0)	0 (0.0)		
Negative	14 (42.4)	8 (40.0)	9 (100.0)		
Metastasis detected on PET/CT					
Hilar and/or mediastinal lymph node metastasis				0.269	0.231
+	13 (39.4)	11 (55.0)	1 (11.1)		
-	20 (60.6)	9 (45.0)	8 (88.9)		
Other site metastasis				0.113	0.112
+	11 (33.3)	2 (10.0)	0 (0.0)		
-	22 (66.7)	18 (90.0)	9 (100.0)		
CT finding					
Polygonal shape				**0.035**	0.135
+	15 (45.5)	15 (75.0)	1 (11.1)		
-	18 (54.5)	5 (25.0)	8 (88.9)		
Ill-defined margin				**0.025**	0.823
+	11 (33.3)	13 (65.0)	4 (44.4)		
-	22 (66.7)	7 (35.0)	5 (55.6)		
Pleural indentation				**0.033**	0.862
+	10 (30.3)	12 (60.0)	3 (33.3)		
-	23 (69.7)	8 (40.0)	6 (66.7)		
Calcification				0.661	0.288
+	3 (9.1)	3 (15.0)	2 (22.2)		
-	30 (90.9)	17 (85)	7 (77.8)		
Cavity				0.09	0.525
+	2 (6.1)	5 (25.0)	1 (11.1)		
-	31 (93.9)	15 (75.0)	8 (88.9)		
Air bronchogram				**0.024**	0.387
+	1 (3.0)	5 (25.0)	1 (11.1)		
-	32 (97.0)	15 (75.0)	8 (88.9)		
Spiculation				**0.031**	0.784
+	13 (39.4)	14 (70.0)	4 (44.4)		
-	20 (60.6)	6 (30.0)	5 (55.6)		
Ground-glass opacity				**0.049**	**0.042**
+	0 (0.0)	3 (15.0)	2 (22.2)		
-	33 (100)	17 (85.0)	7 (77.8)		

SUV: standardized uptake value; PET/CT: positron emission tomography/computed tomography. ^a^Test for difference between the isolated lung metastasis group and the second primary lung cancer group. ^b^Test for difference between the isolated lung metastasis group and the benign nodule group. Statistically significant *p*-values are highlighted in bold.

**Table 3:** Multivariate analysis of the clinical and radiological factors predictive of isolated lung metastasis compared with second primary lung cancer in single lung lesions.

Factors	Risk factor for metastasis	Risk ratio (95% CI)	*p*-value
Gender	Female		0.131
Maximal diameter of lung lesion (mm)	<21.65		0.395
CT finding			
Polygonal shape	—		0.260
Ill-defined margin	—		0.128
Pleural indentation	—		0.308
Air bronchogram	—	22.327 (1.910-261.061)	**0.013**
Spiculation	—	6.148 (1.469-25.725)	**0.013**
Ground-glass opacity	—		0.104

CI: confidence interval. Statistically significant *p*-values are highlighted in bold.

**Table 4:** Univariate and multivariate analysis of factors associated with survival of CRC patients with isolated lung metastasis.

Characteristic	Univariate Analysis HR (95% CI)	*p*-value	Multivariate Analysis HR (95% CI)	*p*-value
Metastasis detected on PET/CT				
Hilar and/or mediastinal lymph node metastasis	6.107 (1.446-25.79)	0.0138	0.605 (0.039-9.342)	0.719
+				
-				
Air bronchogram	29460 (40.01-21690000)	0.0022	0.524 (0.011-25.920)	0.745
+				
-				
Initial TNM stage	680.9 (73.82-6282)	< 0.0001	19.831 (1.061-370.782)	0.046
I-III				
IV				
Treatment of CRC				
Adjuvant chemotherapy	0.2379 (0.06514-0.8688)	0.0298	3.246 (0.248-42.509)	0.37
+				
-				
CA19-9 (U/ml) level	37.98 (3.169-455.3)	0.0041	10.129 (0.470-218.089)	0.139
≥37				
< 37				
ITP (months), median				
≥3/<3 months	0.04844 (0.01041-0.2254)	0.0001	0.016 (0.00-2.529)	0.110
≥6/<6 months	0.04844 (0.01041-0.2254)	0.0001	0.016 (0.00-2.529)	0.110
≥12/<12 months	0.2373 (0.06782-0.8306)	0.0244	2.877 (0.112-73.697)	0.523

CA19-9: carbohydrate antigen 19-9; ITP: interval to pulmonary nodules; CI: confidence interval; HR: hazards ratio.

## Data Availability

The datasets generated or analyzed during the current study are not publicly available in order to preserve patient confidentiality but are available from the corresponding author on reasonable request.

## Supplementary Materials

Supplementary Materials Figure S1 Kaplan-Meier survival analysis of OS in CRC patients with isolated lung metastasis according to initial TNM stage (A), Hilar and/or lymph node metastasis (B), adjuvant chemotherapy of CRC (C), CA19-9 level (D), ITP ≥3/<3 months (E), and ITP ≥12/<12 months (F). The dotted line represents a 50% survival rate. Figure S2.^18^F-FDG PET/CT images of a 69-year-old woman (A-C) and a 57-year-old woman (D-H) with a history of CRC. Maximum-intensity projection (MIP) PET image showed increased ^18^F-FDG uptake in solitary pulmonary lesions (C). The axial image revealed increased ^18^F-FDG uptake (SUV_max_, 6.7; D_max_, 39 mm; A, B) in pulmonary lesions. This patient was still alive at the time of this study with a follow-up time of 23 months. MIP (D), chest axial images (E, F), and abdomen axial images (G, H) of PET/CT images showing increased ^18^F-FDG uptake in the pulmonary lesion (SUV_max_, 3.9; D_max_, 7 mm) and liver lesions. This patient died 9 months after PET/CT scan.
