# Supplementary material for: Imaging Characteristics and Prognostic Value of Isolated Pulmonary Metastasis from Colorectal Cancer Demonstrated with18F-FDG PET/CT
Source: Biomed Res Int. 2022 Apr 14;2022:2230079. doi: 10.1155/2022/2230079 (PMC9023141; doi:10.1155/2022/2230079)
Supplement: Supplementary Materials — Figure S1 Kaplan-Meier survival analysis of OS in CRC patients with isolated lung metastasis according to initial TNM stage (A), Hilar and/or lymph node metastasis (B), adjuvant chemotherapy of CRC (C), CA19-9 level (D), ITP ≥3/<3 months (E), and ITP ≥12/<12 months (F). The dotted line represents a 50% survival rate. Figure S2.18F-FDG PET/CT images of a 69-year-old woman (A-C) and a 57-year-old woman (D-H) with a history of CRC. Maximum-intensity projection (MIP) PET image showed increased 18F-FDG uptake in solitary pulmonary lesions (C). The axial image revealed increased 18F-FDG uptake (SUVmax, 6.7; Dmax, 39 mm; A, B) in pulmonary lesions. This patient was still alive at the time of this study with a follow-up time of 23 months. MIP (D), chest axial images (E, F), and abdomen axial images (G, H) of PET/CT images showing increased 18F-FDG uptake in the pulmonary lesion (SUVmax, 3.9; Dmax, 7 mm) and liver lesions. This patient died 9 months after PET/CT scan. [file 2230079.f1.docx]

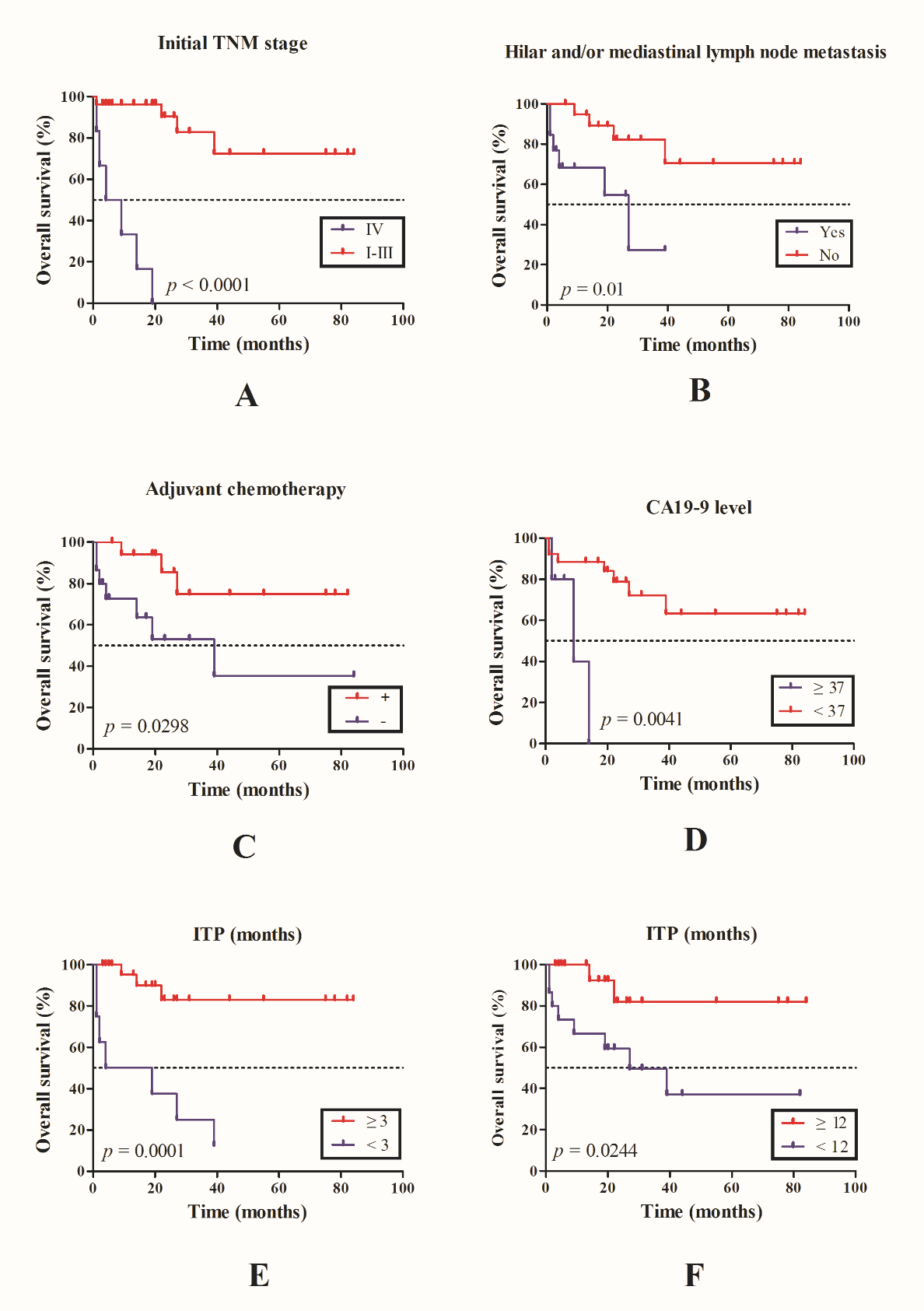


**Figure S1.** Kaplan-Meier survival analysis of OS in CRC patients with isolated lung metastasis according to initial TNM stage (A), mediastinal lymph node metastasis (B), adjuvant chemotherapy of CRC (C), CA19-9 level (D), ITP≥3/<3 months (E), and ITP≥12/<12 months (F). The dotted line represents a 50% survival rate.


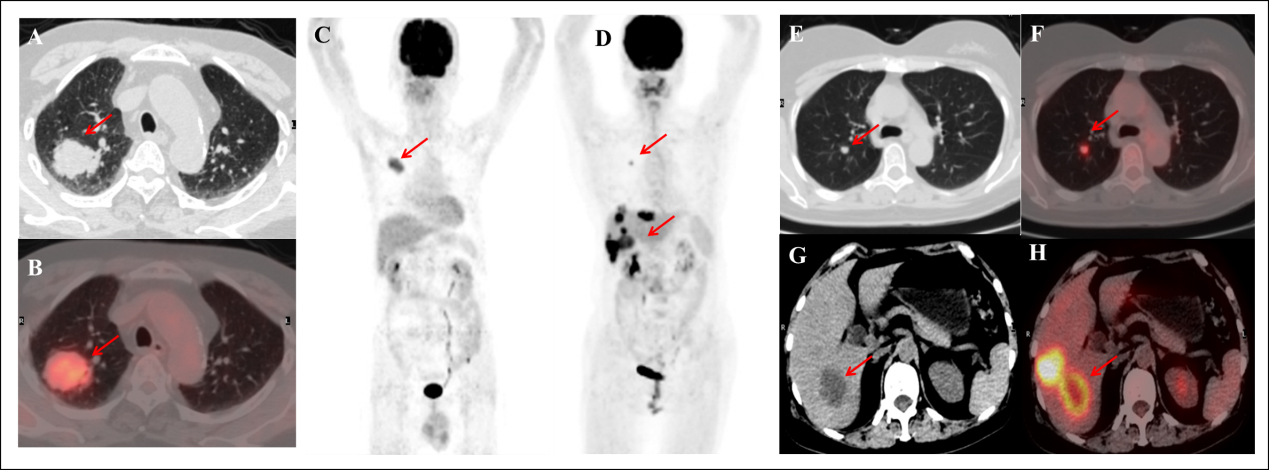


**Figure S2.**^18^F-FDG PET/CT images of a 69-year-old woman (A-C) and a 57-year-old woman (D-H) with a history of CRC. Maximum-intensity projection (MIP) PET image showed increased ^18^F-FDG uptake in solitary pulmonary lesions (C). The axial image revealed increased ^18^F-FDG uptake (SUV_max_, 6.7; D_max_, 39 mm; A, B) in pulmonary lesions. This patient was still alive at the time of this study with a follow-up time of 23 months. MIP (D), chest axial images (E, F), and abdomen axial images (G, H) of PET/CT images showing increased ^18^F-FDG uptake in the pulmonary lesion (SUV_max_, 3.9; D_max_, 7 mm) and liver lesions. This patient died 9 months after PET/CT scan.
